# Efficacy of a midwife-coordinated, individualized, and specialized maternity care intervention (ChroPreg) in addition to standard care in pregnant women with chronic disease: protocol for a parallel randomized controlled trial

**DOI:** 10.1186/s13063-019-3405-5

**Published:** 2019-05-28

**Authors:** Mie Gaarskjaer de Wolff, Marianne Johansen, Anne S. Ersbøll, Susanne Rosthøj, Anne Brunsgaard, Julie Midtgaard, Ann Tabor, Hanne Kristine Hegaard

**Affiliations:** 1 Department of Obstetrics, Copenhagen University Hospital, Rigshospitalet, Blegdamsvej 9, 2100 Copenhagen, Denmark; 2 Research Unit for Women’s and Children’s Health, The Juliane Marie Centre, Copenhagen University Hospital, Rigshospitalet, Blegdamsvej 9, 2100 Copenhagen, Denmark; 3 Faculty of Health and Medical Science, Copenhagen University, Blegdamsvej 3, 2200 Copenhagen, Denmark; 4 Center for Pregnancy and Heart Disease, Copenhagen University Hospital Rigshospitalet, Blegdamsvej 9, Copenhagen, Denmark; 5 Department of Gynecology and Obstetrics, North Zealand Hospital, Dyrehavevej 29, 3400 Hillerød, Denmark; 6 Section of Biostatistics, Department of Public Health, University of Copenhagen, Øster Farimagsgade 5, Entrance B, 2nd floor, Postbox 2099, DK-1014 Copenhagen, Denmark; 7 The University Hospitals Centre for Health Research (UCSF), Copenhagen University Hospital Rigshospitalet, section 9701, Ryesgade 27, 2100 Copenhagen, Denmark; 8 Department of Public Health, University of Copenhagen, Øster Farimagsgade 5, 1014 Copenhagen, Denmark; 9 Center of Fetal Medicine and Pregnancy, Department of Obstetrics, Copenhagen University Hospital, Rigshospitalet, Blegdamsvej 9, 2100 Copenhagen Ø, Denmark; 10 Faculty of Health Sciences, University of Copenhagen, Blegdamsvej 3, 2200 Copenhagen N, Denmark; 11 The Institute of Clinical Medicine, Faculty of Health and Medical Sciences, University of Copenhagen, Blegdamsvej 3, Copenhagen, Denmark

**Keywords:** Pregnancy, Chronic disease, Midwifery, Maternity care, Antenatal care, Psychological well-being, Complex intervention

## Abstract

**Background and objectives:**

The number of women of childbearing age with chronic diseases is rising. Evidence has shown that obstetric complications and poor psychological well-being are more prevalent among this group, in addition to these women reporting experiences of less than satisfactory care. More research is needed to investigate how to best meet the special needs of this group during pregnancy and postpartum. Previous research has shown that care coordination, continuity of care, woman-centered care, and specialized maternity care interventions delivered to women with high-risk pregnancies can improve patient-reported outcomes and pregnancy outcomes and be cost-effective. However, no previous trials have examined the efficacy and cost-effectiveness of such interventions among pregnant women with chronic diseases. This paper describes the protocol of a randomized controlled trial (RCT) of a midwife-coordinated, individualized and specialized maternity care intervention (ChroPreg) as an add-on to standard care for pregnant women with chronic diseases.

**Methods/design:**

This two-arm parallel group RCT will be conducted from October 2018 through June 2020 at the Department of Obstetrics, Copenhagen University Hospital, Rigshospitalet, Denmark. Pregnant women with chronic diseases are invited to participate; women will be randomized and allocated 1:1 to the ChroPreg intervention plus standard care or standard care alone. The ChroPreg intervention consists of three main components: (1) coordinated and individualized care, (2) additional ante- and postpartum consultations, and (3) specialized midwives. The primary outcome is length of hospital stay during pregnancy and in the postpartum period, and secondary outcomes are psychological well-being (five-item World Health Organization Well-Being Index, Edinburgh Postnatal Depression Scale, Cambridge Worry Scale), health-related quality of life (12-Item Short Form Health Survey), patient satisfaction (Pregnancy and Childbirth Questionnaire), number of antenatal contacts, and pregnancy and delivery outcomes. Data are collected via patient-administered questionnaires and medical records.

**Discussion:**

This trial is anticipated to contribute to the field of knowledge on which planning of improved antenatal, intra-, and postpartum care for women with chronic disease is founded.

**Trial registration:**

ClinicalTrials.gov, NCT03511508. Registered April 27, 2018.

**Electronic supplementary material:**

The online version of this article (10.1186/s13063-019-3405-5) contains supplementary material, which is available to authorized users.

## Background

In Denmark and worldwide, the number of individuals living with chronic disease is rising [1, 2], and more than one in three Danish adults live with one or more chronic diseases [1]. The management and prevention of chronic disease is a worldwide major public health problem and one of the issues most targeted by the World Health Organization (WHO) [2].

The number of pregnant women affected by chronic diseases such as autoimmune disease, cardiovascular disease, and endocrine disease is also rising, in 2015, the prevalence in Denmark was 16% among pregnant women [3], and international studies report a prevalence between 16% and 27% [4, 5]. The observed rise in chronic disease among pregnant women reflects the rise in chronic disease in the general population [1]. Factors also contributing to this rise include the increasing age of childbearing women, improvements in medical and surgical treatment options, increased use of assistive reproductive technologies, and improvements in diagnostic challenges with better registration of correct diagnoses [3, 6].

Pregnant women with chronic diseases are a vulnerable group. Overall, these women have a higher risk of preterm birth, pregnancy complications such as gestational diabetes and preeclampsia, operative deliveries, postpartum depression, and longer hospitalization during pregnancy and postpartum [5–9]. In addition to this, children of women with chronic diseases have a higher risk of low birth weight, low Apgar score, and birth defects [10–12]. For some women, their chronic disease may be worsened by pregnancy [13], whereas in other cases of disease, women may experience remission from symptoms during pregnancy [10, 14]. Furthermore, in diseases such as sclerosis and rheumatoid arthritis, there is an increased risk of postpartum flares [14–16].

In qualitative studies, women with chronic diseases indicate that information given by caregivers can be uncoordinated and divergent (e.g., information about medication safety, mode of delivery, and breastfeeding). Women also described less than satisfactory care from midwives and doctors in relation to discussing how to distinguish symptoms of pregnancy and what relates to the disease and to prepare women for what to expect during pregnancy [16]. In addition to these experiences, women described a general experience of “feeling abandoned” postpartum and how support for and understanding of their needs were lacking at the time of discharge [17, 18].

The consequences of the increasing population of pregnant women with chronic diseases are numerous, including a higher demand on maternity care providers to skillfully manage pregnancies complicated by chronic diseases. Furthermore, this increase calls for all maternity care providers to provide coordinated care and consistent counseling [19–21] and to support the pregnant woman in her ability to manage her everyday life with a chronic disease both during pregnancy and postnatally as a new mother [18]. It is therefore important that care providers apply highly specialized medical knowledge combined with an ability to provide psychosocial support in the care of this vulnerable group of pregnant women [6, 16, 22].

Previous randomized controlled trial (RCTs) that evaluated the effect of maternity care interventions for women with high-risk pregnancies have found that the following components had a positive effect on patient-reported outcomes such as patient satisfaction and health economic outcomes such as cost reduction and reduction in length of hospital stay (LOS): implementation of specialized multidisciplinary teams [20, 21, 23], care coordination [19, 24], continuity of care [25], antenatal and postpartum telephone-based support [26], and maternity care with focus on care transitions [22]. In a review of RCTs [22], the introduction of nurse specialist care during pregnancy and the postpartum period of pregnant women with epilepsy demonstrated a positive effect on satisfaction of care provided and a reduction in LOS. To our knowledge, no previous RCT has evaluated the effect of the implementation of a midwife-coordinated, individualized, and specialized maternity care intervention as an add-on intervention to standard care delivered to pregnant women with chronic diseases.

### Aim

The aim of this RCT, the ChroPreg Trial, is to evaluate the efficacy of a midwife-coordinated, individualized, and specialized maternity care intervention as an add-on to standard care for pregnant women with chronic diseases. The primary outcome is LOS measured in days of hospitalization from study inclusion until 2 weeks after delivery. Secondary outcomes are patient-reported psychological well-being, health-related quality of life, satisfaction with care, antenatal contacts, and pregnancy and delivery outcomes.

### Hypothesis

The trial was designed to evaluate the effect of a midwife-coordinated, individualized, and specialized maternity care intervention provided to pregnant women with chronic diseases. We hypothesize that this complex intervention with all its components will have a positive impact on the self-reported psychological well-being of the participants, increase health-related quality of life and satisfaction with the care provided, and enhance the participants’ ability to cope with everyday life during pregnancy and in the immediate postpartum period. We hypothesize that the sum of these components will reduce the total number of days of hospitalization.

Previous trials with complex maternity care interventions with nurse specialists and midwifery continuity-of-care models have shown a reduction in LOS. Tracy et al. [25] showed a mean reduction in LOS of 0.38 days (95% confidence interval, 0.18–0.56) in the postnatal hospital stay among a population of mixed-risk pregnant women (*n* = 1748). We expect that the reduction in LOS will be greater in this trial with a population of high-risk pregnant women with chronic disease. In addition, we will measure LOS both antenatally and postpartum from the time of inclusion and up until 2 weeks after delivery.

## Methods/design

### Study design

The ChroPreg trial is a two-arm parallel-group investigator-initiated RCT. Participants will be randomized to either a midwife-coordinated, individualized, and specialized maternity care intervention (ChroPreg) as an add-on intervention to standard care or standard care alone with a 1:1 allocation. The trial is designed in accordance with the Consolidated Standards of Reporting Trials (CONSORT) guidelines for RCTs [27], extended in the better reporting of interventions: Template for Intervention Description and Replication (TIDieR) checklist and guide [28] (Additional file 1), and in accordance with the Standard Protocol Items: Recommendations for Interventional Trials (SPIRIT) guidelines for reporting trial protocols [29] (Additional file 2).

### Study setting

The study will take place at the Department of Obstetrics, Copenhagen University Hospital, Rigshospitalet, Copenhagen, Denmark. The department is highly specialized and in addition to serving as a birth facility for women living in the local area of the hospital in Copenhagen, it is a tertiary referral center for a large geographical area. In 2017, 5471 women gave birth at the hospital, and 700 of these women had one or more chronic diseases. Participants will be recruited from among the women attending antenatal care at Rigshospitalet. In Denmark, all women with normal pregnancies receive antenatal care primarily from midwives, whereas pregnant women with chronic disease and complicated pregnancies receive obstetrician-led antenatal care, including consultations with midwives.

### Inclusion and exclusion criteria

The Danish Health Authorities define chronic disease as a prolonged disease or a disease that continually recurs [30]. Eligible participants will be pregnant women with one or more chronic diseases diagnosed before pregnancy and for which the pregnant woman is followed by an obstetrician during pregnancy, aged ≥ 18 years, able to understand spoken and written Danish, give informed consent, pregnant with a single live fetus, and be between weeks 12 and 19 of pregnancy at the time of inclusion. Potential participants will be excluded from the study for the following reasons: multiple pregnancy, substance abuse, psychiatric disease as the only chronic disease, and pregnant women with diabetes and heart disease (these women already receive specialized care programs at Rigshospitalet).

### Recruitment procedure

As a part of the standard antenatal care contacts, women who fulfill the study criteria will be invited to participate in the study by a midwife from the antenatal clinic responsible for the coordination of specialized antenatal care. Eligible participants who verbally consent to receive more information about the study will be given written information and subsequently contacted by telephone by one of the investigators. If the woman is still interested in receiving information about the study, preliminary questions will be answered. Thereafter, the woman is given the choice between having a face-to-face meeting at the hospital or receiving additional information by telephone. At the face-to-face meeting or during the telephone conversation, more detailed verbal information about the study will be provided by one of the investigators, and the potential study candidate will have the opportunity to ask further questions about the study. If the woman decides to participate, written informed consent will be obtained by the investigator, and the baseline questionnaire will be completed. The randomization and allocation to one of the two RCT arms will be carried out by one of the investigators. The choice between mode of receiving information, either by telephone or face-to-face, is done to accommodate women who do not live near the hospital or have other reasons to prefer receiving information by telephone. A Danish RCT study tested the effect of face-to-face versus telephone information when including study participants and found no differences in level of comprehension between the two groups [31].

### Randomization and blinding

After informed consent is obtained, participants are randomized by one of the investigators to either the ChroPreg intervention plus standard care or standard care alone in a ratio of 1:1. The allocation sequence is computer-generated and nonstratified with varying block sizes concealed to the researchers. The allocation will be centrally conducted using the EasyTrial online clinical trial management and randomization system (easytrial.net, Aalborg, Denmark). After allocation, the participants will be informed about the allocation and further plan of the trial.

The specialized midwives (SM) who will deliver the intervention will not be blinded to the allocation, and neither will the participants. However, the research staff who will collect data from medical records and questionnaires will be blinded to treatment allocation, as will the chief investigator (HKH) and the trial statistician (SR).

### Sample size calculation

**Fig. 1:**
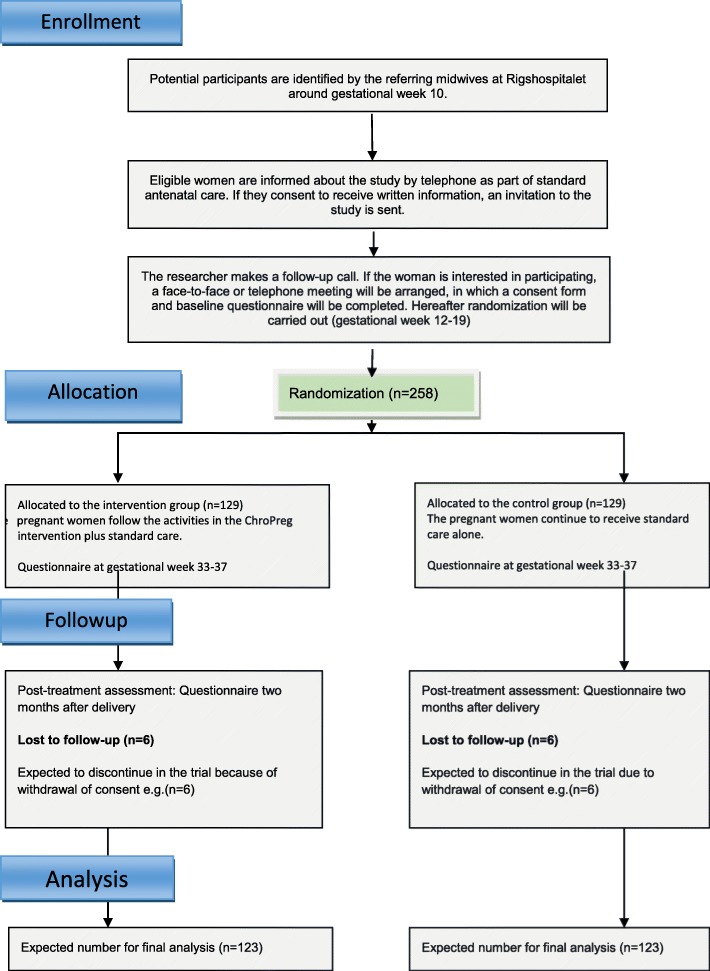
The expected flow diagram of progress through the ChroPreg trial

A total of 258 women will be enrolled in the study. The sample size was determined using unpublished data on 426 pregnant women with chronic diseases hospitalized at Rigshospitalet during pregnancy and in the postpartum period in 2017. The average LOS during pregnancy was 3.9 days; however, due to a right-skewed distribution, log-transformed LOS was used as the outcome for the sample size calculation. Therefore, the effect of the intervention is expressed as a ratio. We expect a reduction of 25% in LOS in the intervention group compared with the control group. This corresponds approximately to a mean reduction of 1 day of hospitalization and to an improvement of log (1 – 0.25) = − 0.29 on the logarithmic scale, which we consider a realistic and clinically significant effect size. The standard deviation (SD) of number of days in the hospital on a logarithmic scale was 0.80. With a difference of 0.29 and SD of 0.80, a power of 0.8, and a significance level of 0.05, an analysis by two-sample *t* test requires 123 women in each group. We expect a maximum of 5% to be lost to follow-up, leading to a total of 129 women needed to be enrolled in each group (Fig. 1).

### Intervention

#### Standard care alone

Participants allocated to the control group will receive standard care alone. Pregnant women with chronic disease are considered to have high-risk pregnancies and are referred to the appropriate level of specialized maternity care. All pregnant women with chronic disease will be followed by an obstetrician, and an individual care plan is designed considering the character and severity of the disease and the pregnancy-associated risks. Routinely in Denmark, all pregnant women, including pregnant women with chronic diseases, are also followed by midwives during pregnancy. The standard care appointments for midwife consultation are timed approximately around weeks 14–18, 28, 35, 38, and 40 and include physical examinations, discussion about lifestyle, symptoms of normal and complicated pregnancies, breastfeeding, and preparation for delivery. At Rigshospitalet, two auditorium prenatal classes (90 min each) are delivered by a midwife. The classes provide information about what to expect during birth (contractions, pain relief, possible complications, and interventions) and in the puerperium (breastfeeding and family building). After childbirth, women can contact the department and request a conversation about the delivery with a midwife, and women who have experienced severe or unexpected complications may be offered a postnatal consultation with an obstetrician.

#### ChroPreg intervention plus standard care

The ChroPreg intervention is designed on the basis of a review of the literature on complex interventions in maternity care [19–21] and is aimed to fit the needs of women with chronic disease. Complex interventions [32] consist of a number of components that may work independently and interdependently, and it can be difficult to identify the “active ingredient” of the intervention, rendering it even more important to describe the different components, the hypothesized mechanism behind each component, and the interaction between the components [32]. The three main components of the ChroPreg intervention are described in more detail below.

The ChroPreg intervention is provided by SM. In addition to the standard care outlined above, women in the intervention group will receive additional antenatal consultations, postpartum follow-up, and care coordination. Both standard care midwife consultations and the extra consultations will be provided by the same SM (content described below and in Table 1).

**Table 1:** Description of planned midwife activities in the ChroPreg intervention group and standard care group

ChroPreg intervention + standard care	Objective of activity	Standard care alone	Objective of activity
During pregnancy			
*Weeks 14–18* Midwife appointment 30 min Specialized midwife (SM) At the hospital	Medical history taking, follow-up on information from general practitioner	*Weeks 14–18* Midwife appointment 30 min Regular midwife (RM) At the hospital	Medical history taking, follow-up on information from general practitioner
*Weeks 20–24* Midwife appointment 60 min SM At the hospital	Focus on psychological well-being during pregnancy and preparation for breastfeeding. The consultation will be centered on the woman’s experience of pregnancy, physical development, prenatal attachment, the interaction between pregnancy and chronic disease, normal pregnancy signs and symptoms, and thoughts about breastfeeding.		
*Weeks 28–30* ^a^ Midwife appointment 30 min SM At the hospital	Conversation about breastfeeding, rhesus prophylaxis, well-being, and symptoms of pregnancy and chronic disease.	*Weeks 28–30* Midwife appointment 30 min RM At the hospital	Conversation about breastfeeding, rhesus prophylaxis, well-being, and symptoms of pregnancy
*Weeks 30–33* Midwife appointment 60 min SM At the hospital	Focus on individual childbirth preparation and postpartum care. Information about birthing options and pain relief and management. The individual needs and wishes of the woman will be discussed, and an individual birth plan will be made.		
*Weeks 35–36* Midwife appointment 30 min SM At the hospital	Consultation about pregnancy, well-being, symptoms of chronic disease, and the coming birth.	*Weeks 35–36* Midwife appointment 30 min RM At the hospital	Consultation about pregnancy and the coming birth, and well-being.
*Weeks 38–39* Midwife appointment 30 min SM At the hospital	Consultation about pregnancy, well-being, symptoms of chronic disease, the coming birth, and postpartum care and follow-up.	*Weeks 38–39* Midwife appointment 30 min RM At the hospital	Consultation about pregnancy, coming birth, and postpartum care
*Week 40* Midwife appointment 30 min SM At the hospital	Consultation about coming birth, pregnancy, and signs of labor, discussion about induced labor Internal vaginal examination if woman wishes	*Week 40* Midwife appointment 30 min RM At the hospital	Consultation about coming birth, pregnancy, and signs of labor; discussion about induced labor Internal vaginal examination if woman wishes
Postpartum follow-up			
*First and second weeks after discharge from hospital* 30–60 min SM Telephone	Postpartum follow-up on breastfeeding, chronic disease management, medication, and psychological well-being. Coordination with primary health care workers if needed (general practitioner/health visitor/medical specialists).	*First week after discharge from hospital*	The woman can contact the hospital with questions and need for postpartum check-up.
*4–6 weeks postpartum* *60 min* Midwife appointment SM At the hospital/telephone (woman’s choice)	This visit is arranged for the woman to have the opportunity to discuss her experiences of pregnancy and childbirth with the SM. The midwife will have the medical records available, but the focus is on allowing the woman to talk about and elaborate on her experience. If the midwife and the woman identify a need for further counseling, referral can be made to a psychologist.	*After discharge from hospital*	The woman can contact the department for an appointment to discuss experiences of pregnancy and childbirth.
Activities throughout the study period			
*E-mail correspondence* From inclusion to end of study period SM	The woman can contact the SM by e-mail. This contact may concern all relevant issues during pregnancy and postpartum.	*E-mail correspondence*	No e-mail correspondence included. Women can contact the coordinating midwife department with questions and concerns
*Weekly telephone hours* From inclusion to end of study period SM	One day each week, the SM will be available for telephone consultation as a supplement to the planned face-to-face consultations. Participants can call the midwife with questions or concerns. The midwife will evaluate if further visits will be needed in addition to the telephone contacts.	*Telephone correspondence*	Women can contact the coordinating midwife department with questions and concerns.

^a^All standard midwife appointments from week 28 onward include urine testing for glucose and protein and measuring of blood pressure (screening for indicators for gestational diabetes and preeclampsia) and physical examinations (measuring the size, heartbeat, and position of the fetus)

##### Midwife-coordinated and individualized care

The participant will be seen by the same SM throughout the study period. For all women in the intervention group, the number of consultations covered by the SM will be recorded. During the study period, the SM will identify the special needs and wishes of each woman and will discuss the optimal care of the woman with the obstetrician responsible for her medical care. The SM will assist the participants in coordinating antenatal visits to fit their everyday life and to avoid unnecessary hospital visits, as well as in communicating with other care providers who are part of the standard Danish health care system (e.g., general practitioner and health visitor) as well as other health care professionals from other departments at the hospital. The participants can contact the SM by e-mail and during weekly phone hours. If the participant is hospitalized during pregnancy, the SM will contact the woman by phone and arrange an appointment. Assisting the participants when in labor is not a part of the intervention.

##### Additional ante- and postpartum consultations

In addition to standard care, two additional antenatal consultations will be scheduled with the SM at 20–24 weeks and 30–33 weeks of pregnancy to discuss psychological well-being in pregnancy, breastfeeding, individualized preparation for childbirth, and postpartum care. The purpose is to support the woman’s own capacity to handle her pregnancy, childbirth, and everyday life as a new mother with a chronic disease. If the need arises, the SM can refer the woman to a psychologist at the hospital for additional consultation. After birth, the SM follows up with the woman regarding psychological well-being during the planned telephone support postpartum, and 4–6 weeks after birth, a consultation is planned with the SM to discuss the woman’s overall experience and issues important to the woman regarding her pregnancy and delivery.

##### Specialized midwives

Before initiation of the study, the SM will have all attended a 3-day training delivered by medical specialists from the hospital handling the care for pregnant women with various chronic diseases. The training includes medical knowledge on systemic lupus erythematosus, rheumatoid arthritis, lung disease, kidney disease, hypertension, multiple sclerosis, epilepsy and other neurological diseases, inflammatory bowel disease, hematological diseases, and thyroid disease. Additional topics in the training program are prepregnancy counseling, pathophysiology, pregnancy complications, symptoms of disease in relation to pregnancy, obstetric treatment, medication and drug safety, lactation, and interdisciplinary collaboration. The training also includes psychological aspects of pregnancy complicated by chronic disease. Topics covered are psychological development during pregnancy, signs and symptoms of depression and anxiety during pregnancy and postpartum, prenatal attachment, and facilitating conversations about the woman’s experience of childbirth. This part of the training is delivered by midwives with further education in psychology and by clinical psychologists. A manual that outlines the content of all consultations with the SM and topics covered during the consultations will be developed and used as a tool by the SM. To secure intervention fidelity, observations of the SM consultations are carried out by one of the investigators in order to give feedback.

### Outcome measures

**Fig. 2:**
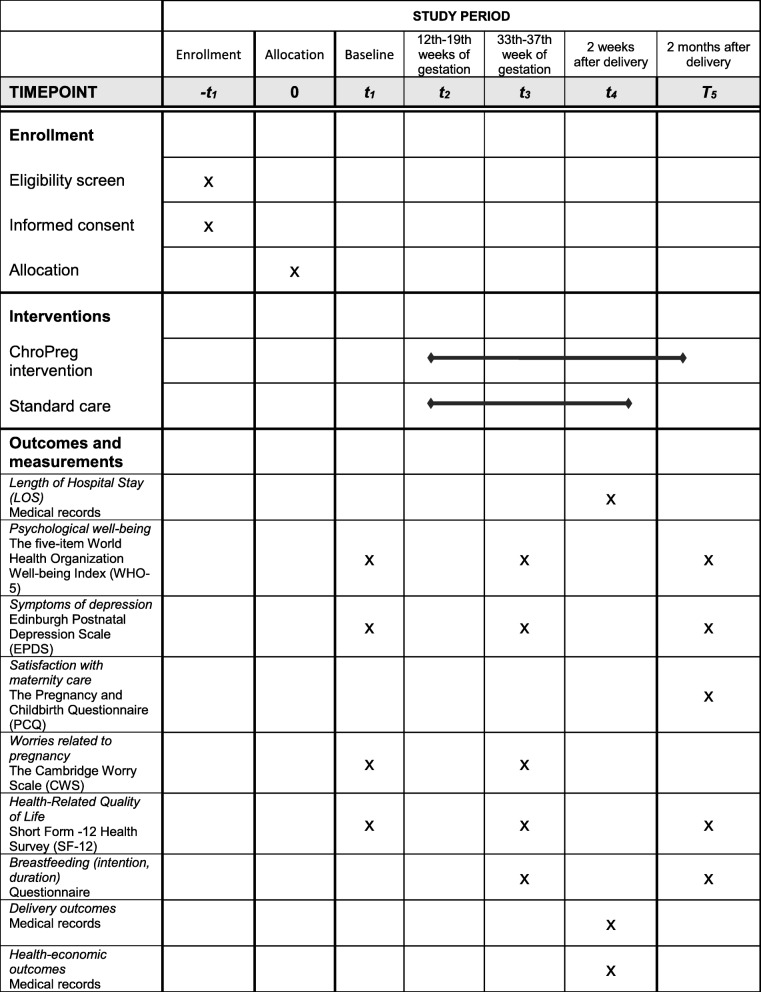
Consolidated Standards of Reporting Trials (CONSORT) diagram showing time points of enrollment, intervention, and outcome measures

Data will be collected from medical records and electronic self-administered questionnaires and will be obtained at baseline (t1) and three times during the study period: at 33–37 weeks of gestation (t3), at 2 weeks after delivery (t4), and at 2 months after delivery (t5) (Fig. 2). The participants will complete the first electronic questionnaire after signing the informed consent to participate and before randomization. The subsequent questionnaires will be sent as a link in an e-mail.

#### Primary outcome

The primary outcome is LOS measured as the number of days (mean and SD) spent in the hospital from inclusion in the study and until 2 weeks after delivery. All participants are expected to have a minimum of 1 day of hospital stay in relation to the delivery, because this population is a high-risk population in which home birth is advised against. Should a woman nevertheless have an unplanned home birth, she will expectedly be transferred to the hospital for postpartum.

LOS was chosen as the primary outcome because it is a widely used outcome in health care research [33, 34]. For this trial, a reduction in LOS covers at least two important outcomes in health care research: (1) the patient perspective with a reduced need for hospitalization, which is an expression of better quality of health care received by the patients, and (2) the health economics perspective with reduced health care costs in connection with days of hospitalization.

#### Secondary outcomes

##### Psychological well-being

1. *Five-item WHO Well-Being Index (WHO-5)* [35] measures current (e.g., preceding 2 weeks) psychological well-being and consists of five positively phrased items, each scored on a Likert scale (0 = none of the time, 5 = all the time). The raw score ranges from 0, indicating the lowest possible well-being, to 25, indicating the highest possible well-being. This score is multiplied by a factor of 4, creating a score ranging from 0 to 100, with 100 being the highest possible well-being. WHO-5 has high psychometric validity, and it can be used as an outcome measure in clinical trials [35].
2. *Edinburgh Postnatal Depression Scale* (EPDS) [36] is a self-reported scale consisting of ten items scored on a 4-point Likert scale (0–3) concerning intensity of depressive mood and symptoms during the last 7 days (0 indicates absence of depressive mood and/or symptoms, and 3 indicates the worst mood and/or symptoms on a given item). The lowest overall score is 0, and the highest overall score is 30 (cutoff scores included will be ≥ 10 and ≥ 13) [36, 37]. The EPDS has been validated for use as a detection tool for antenatal and postnatal depression [37, 38], and it has been translated into Danish [39].
3. *Cambridge Worry Scale* (CWS) [40] measures pregnant women’s degree of worry during pregnancy. The questionnaire consists of 16 items concerning different areas of possible worry on a 6-point Likert scale (0 = no worry, 5 = highest degree of worry). The scale ranges from 0 to 60, with 60 indicating the highest possible level of worry. CWS has a high internal validity and reliability and has been used in clinical studies including populations of women with high-risk pregnancies [41, 42].

##### Health-related quality of life and self-rated health

*SF-12* [43] is an abridged version of the 36-item Short Form Health Survey developed to measure health-related quality of life and thereby self-rated health in a single item. The instrument is well-validated and reliable and is widely used, including during pregnancy and in the postpartum period [43, 44] . The SF-12 questionnaire consists of 12 items that measure physical and mental health, divided into eight subscales: physical and social functioning, role limitation due to physical and mental health, bodily pain, general health perceptions (self-rated health), vitality, and mental health. The scores derived from the subscales can be calculated in two overall scores—Physical Component Summary and Mental Component Summary—with higher scores indicating better health-related quality of life [43].

##### Patient satisfaction

The *Pregnancy and Childbirth Questionnaire (PCQ)* [45] contains two subscales: (1) pregnancy-related items (18 items) covering two domains (personal treatment and patient education/information) and (2) birth-related items (7 items). The PCQ consists of 25 items rated on a 5-point Likert scale (1 = totally agree, 5 = totally disagree). Higher scores indicate higher patient satisfaction with quality of care. The questionnaire has been rated to have good internal consistency and reliability [46].

##### Health economic outcomes

Antenatal contacts will be measured as number of scheduled and unscheduled visits with obstetric doctors and midwives and telephone consultations.

##### Pregnancy and delivery outcomes

Pregnancy and delivery outcomes will be measured as pregnancy complications (gestational diabetes, pregnancy-induced hypertension, preeclampsia) (yes/no), labor onset (spontaneous or induced), preterm delivery (yes/no), mode of delivery (percentage of participants with spontaneous vaginal delivery or cesarean section [planned or emergency]), use of labor analgesia (no analgesia/any analgesia, epidural analgesia [yes/no]), outpatient telemonitoring for pregnancy complications (yes/no and duration [days]), newborn’s well-being at time of delivery (Apgar score ≤ 7 at 5 min postpartum [yes/no]), newborn’s birth weight (measured in kilograms), breastfeeding (intention to breastfeed [yes/no], breastfeeding 2 months after delivery [yes/no], and intended duration [in months] of breastfeeding).

### Data analysis

Analyses are to be performed on the basis of the intention-to-treat principle. The log-transformed number of days in the hospital in the two groups will be compared in the primary unadjusted analysis, and in a secondary analysis, adjustments will be made for parity and age. Analysis will be performed using the general linear model unless we find a generalized linear model (e.g., Poisson or negative binomial) to fit the distribution of the number of days in the hospital. Furthermore, the two groups will be compared with respect to the proportion of women with 3 or fewer days in the hospital. Comparison of secondary and exploratory outcomes will similarly be performed using the general or generalized linear models as appropriate. In case of an uneven allocation of pregnancies with congenital malformations or chromosomal abnormalities to the two groups, analyses will be performed in the subgroup of women without pregnancies with congenital malformations or chromosomal abnormalities.

## Discussion

The WHO estimates that chronic disease is the cause of the majority of premature deaths in the world and that most of these could be prevented by giving people better access to appropriate health care and treatment and by reducing modifiable risk factors such as diet, exercise, and smoking [2]. Worldwide, the proportion of women of childbearing age living with one or more chronic diseases is growing [3, 5], and pregnancies affected by chronic disease are associated with a higher risk of complications [6, 10] and poor psychological health [8, 47].

The perinatal period is theoretically a favorable time in a woman’s life to implement effective preventive and health-promoting interventions in women [48]. Interventions delivered in the perinatal period can therefore hypothetically reduce the potentially negative health impact in pregnancy caused by chronic disease and possibly also empower and strengthen the women’s long-term self-care ability with their chronic disease.

The ChroPreg trial is highly relevant, and to the best of our knowledge, it is the largest RCT designed to investigate the effect of a midwife-coordinated, individualized, and specialized maternity care add-on intervention for pregnant women with chronic diseases on LOS, patient-reported outcomes, and pregnancy and delivery outcomes.

Systematic reviews of RCTs have found previous complex maternity care interventions to be cost-effective compared with standard care [24, 25]. However, in these reviews, the authors have called for trials that evaluate psychological well-being and patient satisfaction with maternity care with validated psychometric instruments [19, 49]. Therefore, in this RCT, we will use validated patient-reported questionnaires to estimate psychological well-being during pregnancy and postpartum [35, 36, 40], health-related quality of life [43], and patient satisfaction with maternity care [45].

In this trial, we will also combine face-to-face consultations with add-on telephone support and e-mail correspondence delivered by the SM who follow the participants throughout pregnancy and postpartum. Telephone support during pregnancy and after birth has been suggested to be of benefit to women at risk of depression, reducing signs of depression and facilitating breastfeeding initiation and continuation [26]. However, these interventions have not yet been tested among pregnant women with chronic diseases, and therefore the results of this trial may provide information on how to design future woman-centered and cost-effective interventions for women with chronic disease. This trial will also provide information to clinicians about the feasibility of prolonging midwifery care with consultations and more thorough follow-up after discharge from the hospital, because this care currently is delivered primarily by health visitors in Denmark.

Because this is a low-risk intervention adding to standard care and not involving any known side effects, we anticipate that most eligible women with chronic disease will be interested in participating in the trial, and we do not expect a high number of dropouts. Measures to monitor and enhance adherence to the intervention will be taken. The two self-reported questionnaires, one at 33–37 weeks of pregnancy and one at 2 months postpartum, will be followed by reminders if not completed within the time frame, and text message reminders will be sent in advance; adherence to all planned activities of the interventions will be monitored and recorded. The primary outcome (LOS) data are collected from medical records and do not rely on patient self-reporting. To reduce the risk of selection bias, eligible women will be offered the choice between a face-to-face meeting and a telephone conversation for information about the study, informed consent, and randomization, accommodating women living farther from the hospital.

The ChroPreg trial will provide an extensive evaluation of a coordinated, individualized, and specialized maternity care intervention for pregnant women with chronic diseases. This trial will be among the first of its kind and will help clinicians and policy makers to make evidence-based decisions when developing and implementing efficient, woman-centered, and cost-effective maternity care interventions for women with chronic disease.

To conclude, this paper outlines the background, aim, and methods of an RCT in which the ChroPreg intervention plus standard care is compared with standard care alone in a population of pregnant women with chronic disease. We hypothesize that the sum of active components in the ChroPreg intervention will reduce the overall need for hospitalization and enhance the psychological well-being and satisfaction with care for the participants.

## Trial status

Recruitment of participants was initiated October 1, 2018, and is anticipated to continue until January 31, 2020, and data collection will be concluded by June 1, 2020. By May 22 2019, 130 participants have been included in the Trial. No interim analyses will be performed.

## Additional files

Additional file 1: TIDieR checklist. (PDF 447 kb)

Additional file 2: SPIRIT checklist. (DOC 122 kb)

## Data Availability

The datasets used and/or analyzed during the current study are available from the corresponding author on reasonable request.

## Abbreviations

CONSORT: Consolidated Standards of Reporting Trials
CWS: Cambridge Worry Scale
EPDS: Edinburgh Postnatal Depression Scale
GP: General practitioner
LOS: Length of hospital stay
PCQ: Pregnancy and Childbirth Questionnaire
RM: Regular midwife
SD: Standard deviation
SM: Specialized midwife
SPIRIT: Standard Protocol Items: Recommendations for Interventional Trials
TIDieR: Template for Intervention Description and Replication
WHO-5: World Health Organization Well-Being Index

## References

[1] Danish Health Authorities IJS (2005). Kronisk sygdom. Patient, sundhedsvæsen og samfund. Chronic disease. Patient, healthcare system and society. Copenhagen: Danish Health and Medicines Authority.

[2] World Health Organization (WHO) (2017). Noncommunicable diseases progress monitor, 2017.

[3] Jolving LR, Nielsen J, Kesmodel US, Nielsen RG, Beck-Nielsen SS, Norgard BM (2016). Prevalence of maternal chronic diseases during pregnancy - a nationwide population based study from 1989 to 2013. Acta Obstet Gynecol Scand.

[4] Chatterjee S, Kotelchuck M, Sambamoorthi U (2008). Prevalence of chronic illness in pregnancy, access to care, and health care costs: implications for interconception care. Womens Health Issues.

[5] Kersten I, Lange AE, Haas JP, Fusch C, Lode H, Hoffmann W (2014). Chronic diseases in pregnant women: prevalence and birth outcomes based on the SNiP-study. BMC Pregnancy Childbirth.

[6] Narayan B, Jarvis S, Dassan P, Nelson-Piercy C (2018). Medical problems in pregnancy. Clin Med (Lond).

[7] Jolving LR, Nielsen J, Beck-Nielsen SS, Nielsen RG, Friedman S, Kesmodel US (2017). The association between maternal chronic inflammatory bowel disease and long-term health outcomes in children—a nationwide cohort study. Inflamm Bowel Dis.

[8] Bjork MH, Veiby G, Reiter SC, Berle JO, Daltveit AK, Spigset O (2015). Depression and anxiety in women with epilepsy during pregnancy and after delivery: a prospective population-based cohort study on frequency, risk factors, medication, and prognosis. Epilepsia..

[9] Bendix J, Hegaard HK, Langhoff-Roos J, Bergholt T (2016). Changing prevalence and the risk factors for antenatal obstetric hospitalizations in Denmark 2003-2012. Clin Epidemiol.

[10] Bramham K, Parnell B, Nelson-Piercy C, Seed PT, Poston L, Chappell LC (2014). Chronic hypertension and pregnancy outcomes: systematic review and meta-analysis. BMJ..

[11] Viale L, Allotey J, Cheong-See F, Arroyo-Manzano D, McCorry D, Bagary M (2015). Epilepsy in pregnancy and reproductive outcomes: a systematic review and meta-analysis. Lancet.

[12] Smyth A, Oliveira GH, Lahr BD, Bailey KR, Norby SM, Garovic VD (2010). A systematic review and meta-analysis of pregnancy outcomes in patients with systemic lupus erythematosus and lupus nephritis. Clin J Am Soc Nephrol..

[13] Jara LJ, Medina G, Cruz-Dominguez P, Navarro C, Vera-Lastra O, Saavedra MA (2014). Risk factors of systemic lupus erythematosus flares during pregnancy. Immunol Res.

[14] de Man YA, Dolhain RJ, Hazes JM (2014). Disease activity or remission of rheumatoid arthritis before, during and following pregnancy. Curr Opin Rheumatol.

[15] Bove R, Alwan S, Friedman JM, Hellwig K, Houtchens M, Koren G (2014). Management of multiple sclerosis during pregnancy and the reproductive years: a systematic review. Obstet Gynecol.

[16] de Wolff M, Ersboll AS, Hegaard H, Johansen M, Gustafsson F, Damm P (2018). Psychological adaptation after peripartum cardiomyopathy: a qualitative study. Midwifery..

[17] Thomas H (2004). Women’s postnatal experience following a medically complicated pregnancy. Health Care Women Int.

[18] Weckesser A, Denny E (2013). Women living with epilepsy, experiences of pregnancy and reproductive health: a review of the literature. Seizure..

[19] Kroll-Desrosiers AR, Crawford SL, Moore Simas TA, Rosen AK, Mattocks KM (2016). Improving pregnancy outcomes through maternity care coordination: a systematic review. Womens Health Issues.

[20] Schmied V, Mills A, Kruske S, Kemp L, Fowler C, Homer C (2010). The nature and impact of collaboration and integrated service delivery for pregnant women, children and families. J Clin Nurs.

[21] Rodriguez C, des Rivieres-Pigeon C (2007). A literature review on integrated perinatal care. Int J Integr Care.

[22] Bryant-Lukosius D, Carter N, Reid K, Donald F, Martin-Misener R, Kilpatrick K (2015). The clinical effectiveness and cost-effectiveness of clinical nurse specialist-led hospital to home transitional care: a systematic review. J Eval Clin Pract.

[23] Kehlet H, Wilmore DW (2008). Evidence-based surgical care and the evolution of fast-track surgery. Ann Surg.

[24] Little M, Saul GD, Testa K, Gaziano C (2002). Improving pregnancy outcome and reducing avoidable clinical resource utilization through telephonic perinatal care coordination. Lippincotts Case Manag.

[25] Tracy SK, Hartz DL, Tracy MB, Allen J, Forti A, Hall B (2013). Caseload midwifery care versus standard maternity care for women of any risk: M@NGO, a randomised controlled trial. Lancet.

[26] Lavender T, Richens Y, Milan SJ, Smyth RM, Dowswell T (2013). Telephone support for women during pregnancy and the first six weeks postpartum. Cochrane Database Syst Rev.

[27] Schulz KF, Altman DG, Moher D (2011). CONSORT 2010 statement: updated guidelines for reporting parallel group randomised trials. Int J Surg.

[28] Hoffmann TC, Glasziou PP, Boutron I, Milne R, Perera R, Moher D (2016). Better reporting of interventions: Template for Intervention Description and Replication (TIDieR) checklist and guide [in German]. Gesundheitswesen.

[29] Chan AW, Tetzlaff JM, Altman DG, Laupacis A, Gøtzsche PC, Krle A-Jerić K (2015). SPIRIT 2013 statement: defining standard protocol items for clinical trials. Rev Panam Salud Publica.

[30] Sundhedsstyrelsen DHA (2012). Den generiske model for forløbsprogrammer for kronisk sygdom.

[31] Foss KT, Kjaergaard J, Stensballe LG, Greisen G (2016). Recruiting to clinical trials on the telephone - a randomized controlled trial. Trials.

[32] Medical Research Council (2006). Developing and evaluating complex interventions: new guidance.

[33] Lingsma HF, Bottle A, Middleton S, Kievit J, Steyerberg EW, Marang-van de Mheen PJ (2018). Evaluation of hospital outcomes: the relation between length-of-stay, readmission, and mortality in a large international administrative database. BMC Health Serv Res.

[34] Campbell OM, Cegolon L, Macleod D, Benova L (2016). Length of stay after childbirth in 92 countries and associated factors in 30 low- and middle-income countries: compilation of reported data and a cross-sectional analysis from nationally representative surveys. PLoS Med.

[35] Topp CW, Ostergaard SD, Sondergaard S, Bech P (2015). The WHO-5 Well-Being Index: a systematic review of the literature. Psychother Psychosom.

[36] Cox JL, Holden JM, Sagovsky R (1987). Detection of postnatal depression: development of the 10-item Edinburgh Postnatal Depression Scale. Br J Psychiatry.

[37] Glaze R, Cox JL (1991). Validation of a computerised version of the 10-item (self-rating) Edinburgh Postnatal Depression Scale. J Affect Disord.

[38] Tendais I, Costa R, Conde A, Figueiredo B (2014). Screening for depression and anxiety disorders from pregnancy to postpartum with the EPDS and STAI. Span J Psychol.

[39] Nielsen Forman D, Videbech P, Hedegaard M, Dalby Salvig J, Secher NJ (2000). Postpartum depression: identification of women at risk. BJOG.

[40] Green JM, Kafetsios K, Statham HE, Snowdon CM (2003). Factor structure, validity and reliability of the Cambridge Worry Scale in a pregnant population. J Health Psychol.

[41] Asplin N, Wessel H, Marions L, Georgsson Öhman S (2015). Maternal emotional wellbeing over time and attachment to the fetus when a malformation is detected. Sex Reprod Healthc.

[42] Petersen JJ, Paulitsch MA, Guethlin C, Gensichen J, Jahn A (2009). A survey on worries of pregnant women - testing the German version of the Cambridge Worry Scale. BMC Public Health.

[43] Loosman WL, Hoekstra T, van Dijk S, Terwee CB, Honig A, Siegert CE (2015). Short-Form 12 or Short-Form 36 to measure quality-of-life changes in dialysis patients?. Nephrol Dial Transplant.

[44] Vinturache A, Stephenson N, McDonald S, Wu M, Bayrampour H, Tough S (2015). Health-related quality of life in pregnancy and postpartum among women with assisted conception in Canada. Fertil Steril.

[45] Truijens SE, Pommer AM, van Runnard Heimel PJ, Verhoeven CJ, Oei SG, Pop VJ (2014). Development of the Pregnancy and Childbirth Questionnaire (PCQ): evaluating quality of care as perceived by women who recently gave birth. Eur J Obstet Gynecol Reprod Biol..

[46] Nilver H, Begley C, Berg M (2017). Measuring women’s childbirth experiences: a systematic review for identification and analysis of validated instruments. BMC Pregnancy Childbirth..

[47] Brown HK, Qazilbash A, Rahim N, Dennis CL, Vigod SN (2018). Chronic medical conditions and peripartum mental illness: a systematic review and meta-analysis. Am J Epidemiol.

[48] Haakstad LA, Voldner N, Bo K (2013). Stages of change model for participation in physical activity during pregnancy. J Pregnancy.

[49] Sandall J, Soltani H, Gates S, Shennan A, Devane D (2015). Midwife-led continuity models versus other models of care for childbearing women. Cochrane Database Syst Rev.

[50] Komitéloven. Bekendtgørelse af lov om videnskabsetisk behandling af sundhedsvidenskabelige forskningsprojekter. LBK no. 1083. 15 Sep 2017.

[51] General Assembly of the World Medical Association (2014). World Medical Association Declaration of Helsinki: ethical principles for medical research involving human subjects. J Am Coll Dentists.

